# Identification of *Eleutherococcus senticosus* NAC transcription factors and their mechanisms in mediating DNA methylation of *EsFPS*, *EsSS*, and *EsSE* promoters to regulate saponin synthesis

**DOI:** 10.1186/s12864-024-10442-8

**Published:** 2024-05-31

**Authors:** Jing Dong, Xuelei Zhao, Xin Song, Shuo Wang, Xueying Zhao, Baoxiang Liang, Yuehong Long, Zhaobin Xing

**Affiliations:** College of Life Sciences, North China University of Science and Technology, Tangshan, 063210 Hebei China

**Keywords:** *Eleutherococcus senticosus*, DNA methylation, NAC transcription factors, Key enzyme genes

## Abstract

**Background:**

The formation of pharmacologically active components in medicinal plants is significantly impacted by DNA methylation. However, the exact mechanisms through which DNA methylation regulates secondary metabolism remain incompletely understood. Research in model species has demonstrated that DNA methylation at the transcription factor binding site within functional gene promoters can impact the binding of transcription factors to target DNA, subsequently influencing gene expression. These findings suggest that the interaction between transcription factors and target DNA could be a significant mechanism through which DNA methylation regulates secondary metabolism in medicinal plants.

**Results:**

This research conducted a comprehensive analysis of the NAC family in *E. senticosus*, encompassing genome-wide characterization and functional analysis. A total of 117 *EsNAC* genes were identified and phylogenetically divided into 15 subfamilies. Tandem duplications and chromosome segment duplications were found to be the primary replication modes of these genes. Motif 2 was identified as the core conserved motif of the genes, and the cis-acting elements, gene structures, and expression patterns of each *EsNAC* gene were different. *Es*JUB1, *Es*NAC047, *Es*NAC098, and *Es*NAC005 were significantly associated with the DNA methylation ratio in *E. senticosus.* These four genes were located in the nucleus or cytoplasm and exhibited transcriptional self-activation activity. DNA methylation in *EsFPS*, *EsSS*, and *EsSE* promoters significantly reduced their activity. The methyl groups added to cytosine directly hindered the binding of the promoters to *Es*JUB1, *Es*NAC047, *Es*NAC098, and *Es*NAC005 and altered the expression of *EsFPS*, *EsSS*, and *EsSE* genes, eventually leading to changes in saponin synthesis in *E. senticosus.*

**Conclusions:**

NAC transcription factors that are hindered from binding by methylated DNA are found in *E. senticosus*. The incapacity of these NACs to bind to the promoter of the methylated saponin synthase gene leads to subsequent alterations in gene expression and saponin synthesis. This research is the initial evidence showcasing the involvement of *Es*NAC in governing the impact of DNA methylation on saponin production in *E. senticosus*.

**Supplementary Information:**

The online version contains supplementary material available at 10.1186/s12864-024-10442-8.

## Background

*Eleutherococcus senticosus* is a significant and traditional medicinal plant in China [1], known for its triterpenoid saponins as one of its active constituents [2]. To date, various products of *E. senticosus* have been commercialized as clinical drugs or dietary supplements in East Asia, Europe, and the United States of America [3, 4]. The demand for products related to *E. senticosus* in the consumer market is increasing. However, the reproductive capacity of *E. senticosus* is weak under natural conditions, leading to its serious shortage [5]. Therefore, understanding the molecular mechanisms that determine the content of active components in *E. senticosus* is important for realizing the sustainable utilization of *E. senticosus*.

These molecular mechanisms can be determined by analyzing changes at the DNA level in *E. senticosus*. Saponins are synthesized from isopentenyl diphosphate (IPP) and dimethyl allyl diphosphate (DMAPP) in *E. senticosus* [6]. Farnesyl diphosphate (FPP) is formed through the 1,4 head-to-tail condensation of two IPP molecules and one DMAPP molecule, catalyzed by farnesyl diphosphate synthase (FPS). FPP can provide a C15 skeleton for many metabolites and is a common precursor for the synthesis of terpenoids, steroids, and sterols. Squalene synthase (SS) catalyzes the synthesis of squalene from two molecules of FPP. Following that, squalene undergoes oxidation to produce 2,3-squalene oxide with the assistance of squalene epoxidase (SE) and is then transformed into various triterpene skeletons through cyclization [7, 8]. *EsFPS*, *EsSS*, and *EsSE* are three crucial enzymes that play a significant role in the synthesis of triterpenoid saponins [9]. Research has indicated that the complete understanding of the saponin content in *E. senticosus* cannot be achieved solely through traditional genetic analysis methods like SNPs and CNVs of *EsFPS*, *EsSS*, and *EsSE* genes [8]. This implies that genetic mechanisms other than DNA sequence variations may play an important role.

A study found that DNA methylation sites and levels in the promoters of *EsFPS*, *EsSS*, *EsSE* [8]and mevalonate pyrophosphate decarboxylase (MDD) [10] genes in *E. senticosus*, had an impact on gene expression and saponin synthesis. These factors play a crucial role in determining the synthesis and quality of saponins [8, 10]. The discovery suggest that DNA methylation plays a significant role in the alteration of key enzyme-coding genes’ expression and the enhancement of active components’ quality in *E. senticosus* [8, 11]. Nonetheless, the precise mechanisms responsible for alterations in DNA methylation within these genes in medicinal plants are still not fully understood [12].

Studies on humans and model organisms at the molecular level have shown that DNA methylation cannot directly exert biological effects, such as regulating gene expression. Instead, its role depends on two non-exclusive pathways [13]. First, methylated DNA is recognized by proteins that specifically recognize 5-mC [14] and recruit other proteins to form a transcription inhibition complex, indirectly inducing histone deacetylation, altering the structure of DNA and chromosomes, and leading to inhibition of transcription [15, 16]. Furthermore, the presence of 5-mC has an impact on the interaction between transcription factors (TFs) and this specific location, ultimately resulting in alterations in gene expression [17–19]. The second route has a greater impact on regulation. For example, DNA methylation in rice affects the binding between TFs and TF-binding sites (TFBSs), leading to changes in gene expression and related traits [20]. Therefore, screening and identifying transcription factors that are sensitive to methylated DNA and elucidating the biological effects resulting from the binding of TFs to TFBSs in functional gene promoters are potential approaches to analyzing the regulatory effects of DNA methylation on the content of saponins in *E. senticosus*.

NAC transcription factors (TFs) are highly prevalent regulators of gene expression in plants, and they play an important role in the development of plant organs and their ability to adapt to environmental stresses and viral infections [21]. The term NAC originates from the designations of three genes found in the petunia apical meristem (NAM), ATAF1/2, and *Arabidopsis thaliana* cup cotyledon 2 (CUC2) genes [22]. In general, the N-terminus of NAC usually possesses a preserved NAC domain that has the ability to bind to DNA and additional proteins. On the other hand, the C-terminus consists of a variable transcription activation region (TAR) that can either activate or hinder gene transcription [23]. Recent studies on tomato have found that NAC immature transcription factor (NAC-NOR) can activate the expression of DNA demethylase 2 (SlDML2) by directly binding to its promoter both in vitro and in vivo. The decrease in NAC-NOR expression in SLDML2 mutants is accompanied by hypermethylation of its promoter [24]. NAC050 and NAC052 in *Arabidopsis thaliana* have the ability to engage in histone demethylation, contributing significantly to the regulation of RNA-mediated gene silencing and flowering time [25]. Therefore, NAC is involved in several processes, such as DNA methylation, which regulates gene expression in organisms.

NAC TFs have binding sites in the promoters of *EsFPS*, *EsSS*, and *EsSE* in *E. senticosus.* However, the role of *Es*NAC in regulating DNA methylation and secondary metabolism in *E. senticosus* remains unclear. In this study, genome-wide assessment revealed *EsNAC* genes that are sensitive to methylated DNA. Validation of gel block analysis, dual-luciferase reporter gene assay, and overexpression, among others, confirmed that DNA methylation hindered the interaction between *EsNAC* and the promoter of the crucial enzyme-coding gene associated with saponin synthesis in *E. senticosus*. Consequently, this led to alterations in both gene expression and saponin content.

## Results

### Identification of *EsNAC* genes and their localization on chromosomes

**Fig. 1:**
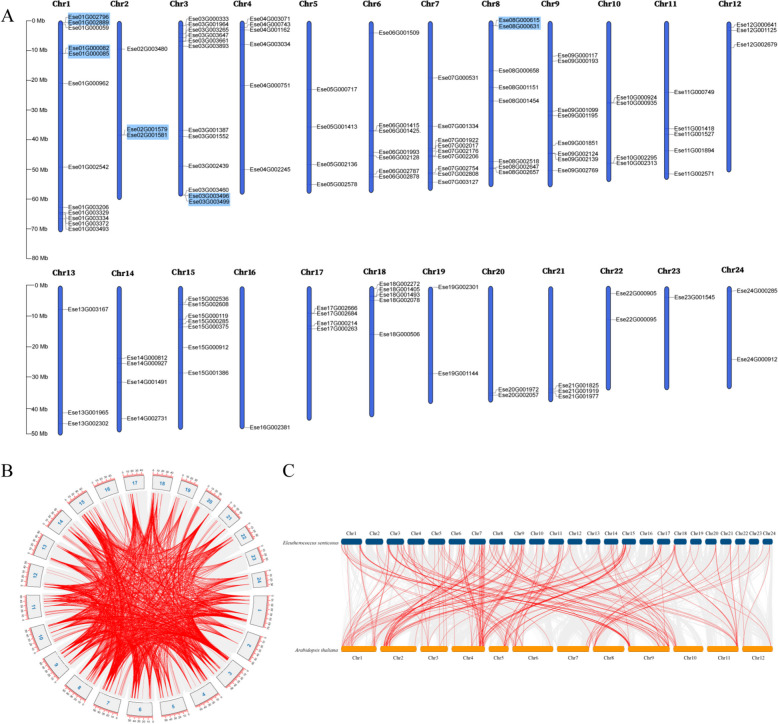
Chromosomal localization and collinearity analysis of *EsNAC* genes. **A** Location information of *EsNAC* genes on chromosomes; genes highlighted in blue represent tandemly duplicated genes. **B** Intraspecies collinearity analysis of *EsNAC* genes. **C** Inter-species collinearityanalysis of *EsNAC* genes in *E. senticosus *and *Aralia elata*. Red indicates collinearity *NAC* genes, and gray indicates other collinearity genes

From the genome sequencing data of *E. senticosus*, a total of 117 *EsNAC* genes were discovered, with lengths ranging from 381 bp to 1866 bp as shown in Supplementary Table 2. The distribution of these genes was not uniform across 24 chromosomes (Fig. 1, A). Specifically, chromosomes 1 and 3 each contained 12 genes, while chromosome 7 contained 9 genes, chromosome 8 contained 8 genes, and chromosomes 16 and 23 each contained 1 gene. The *EsNAC* genes mostly existed in the form of clusters on chromosomes, often distributed at both ends of the chromosomes. Five pairs of tandemly duplicated genes were found in these clusters. These results suggest that multiple gene duplication events occur in the *EsNAC* gene family and tandem duplication is an important mechanism for the evolution of *EsNAC* genes, which affects the evolution of *NAC* genes in *E. senticosus*.

### Co-linearity analysis of the *Es*NAC gene family

A total of 3160 collinear *EsNAC* gene pairs were identified in *E. senticosus*. These genes were unevenly distributed on 24 chromosomes (Fig. 1, B), accounting for 0.53% of all collinear genes in *E. senticosus*. The genes exhibited interchromosomal and intrachromosomal covariance. Among them chromosome 23 had the lowest number of interchromosomal collinear *EsNAC* genes (21), accounting for 0.67% of all genes, whereas chromosome 1 had the largest number of interchromosomal collinear *EsNAC* genes (436), accounting for 13.8% of all genes. These results suggest that chromosome segment duplication may have occurred during the evolution of the *EsNAC* gene family.

To examine the evolutionary relationship of the *NAC* gene family between *E. senticosus* and its relatives, we selected *A. elata*, whose genome has been completely sequenced, for inter-species collinearity analysis. A total of 46,288 pairs of inter-species collinear genes were identified; of which, 205 pairs were co-lineage *NAC* genes between *E. senticosus* and *A. elata*, accounting for 0.443% of all genes (Fig. 1 C). The higher distribution of interspecies collinear *NAC* genes on chromosomes 3, 7, and 9 in *E. senticosus* indicated that the two species shared more homologous genes, were more closely related. The *Ese13G001965* on chromosome 13 of *E. senticosus* has collinear relationships with *AE01G00187* on chromosome 1, *AE09G03799* on chromosome 9, and *AE10G00689* on chromosome 10 of *A. elata*, indicating that *NAC* genes have undergone more than one replication events. Moreover, the collinear genes of *E. senticosus* and *A. elata* were distributed in clusters at both ends of chromosomes, indicating that the gene replication patterns of *E. senticosus* and *A. elata*, a close relative of *E. senticosus*, were similar.

### Phylogenetic analysis of the NAC gene family

**Fig. 2:**
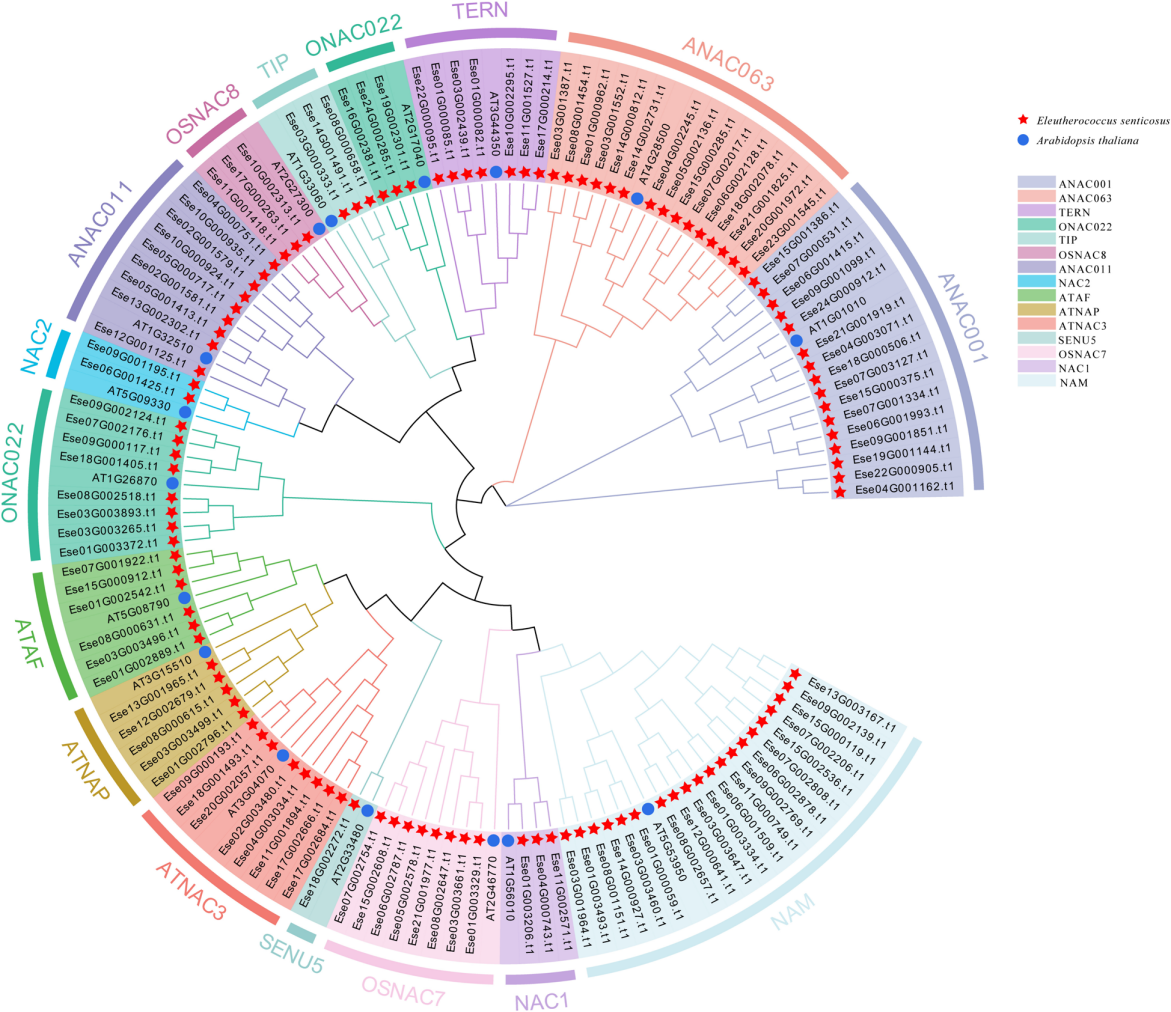
Phylogenetic analysis of NAC transcription factors in *E. senticosus*

The 16 NAC genes that have been typed in *A. thaliana* and the 117 *Es*NAC proteins identified in this study were selected for classification and phylogenetic analysis with reference to the methods described by Ooka et al. [22] and Pei et al. [26]. NAC genes were categorized into 15 subfamilies, with the number of genes in each subfamily being different (Fig. 2). In particular, the NAM subfamily had the highest number of NAC genes (20 genes), followed by the ANAC001 subfamily (16 genes), whereas the SENU5 subfamily contained only 1 NAC gene. It is noteworthy that the *EsNAC* gene of the ONAC022 subfamily was divided into two parts by other subfamilies, which was different from the subfamily distribution of NAC genes in *Oryza sativa L* [22]. The tandemly duplicated genes identified were unevenly distributed in subfamilies. The tandemly duplicated gene pairs on chromosome 8 were assigned to two subfamilies. Ese08G000615.t1 was assigned to the ATNAP subfamily, whereas Ese08G000631.t1 was assigned to the ATAF subfamily. These results indicate that the more closely related NAC genes may have produced different functional differentiation patterns during evolution.

### Motif and gene structure analysis of *EsNAC* genes

**Fig. 3:**
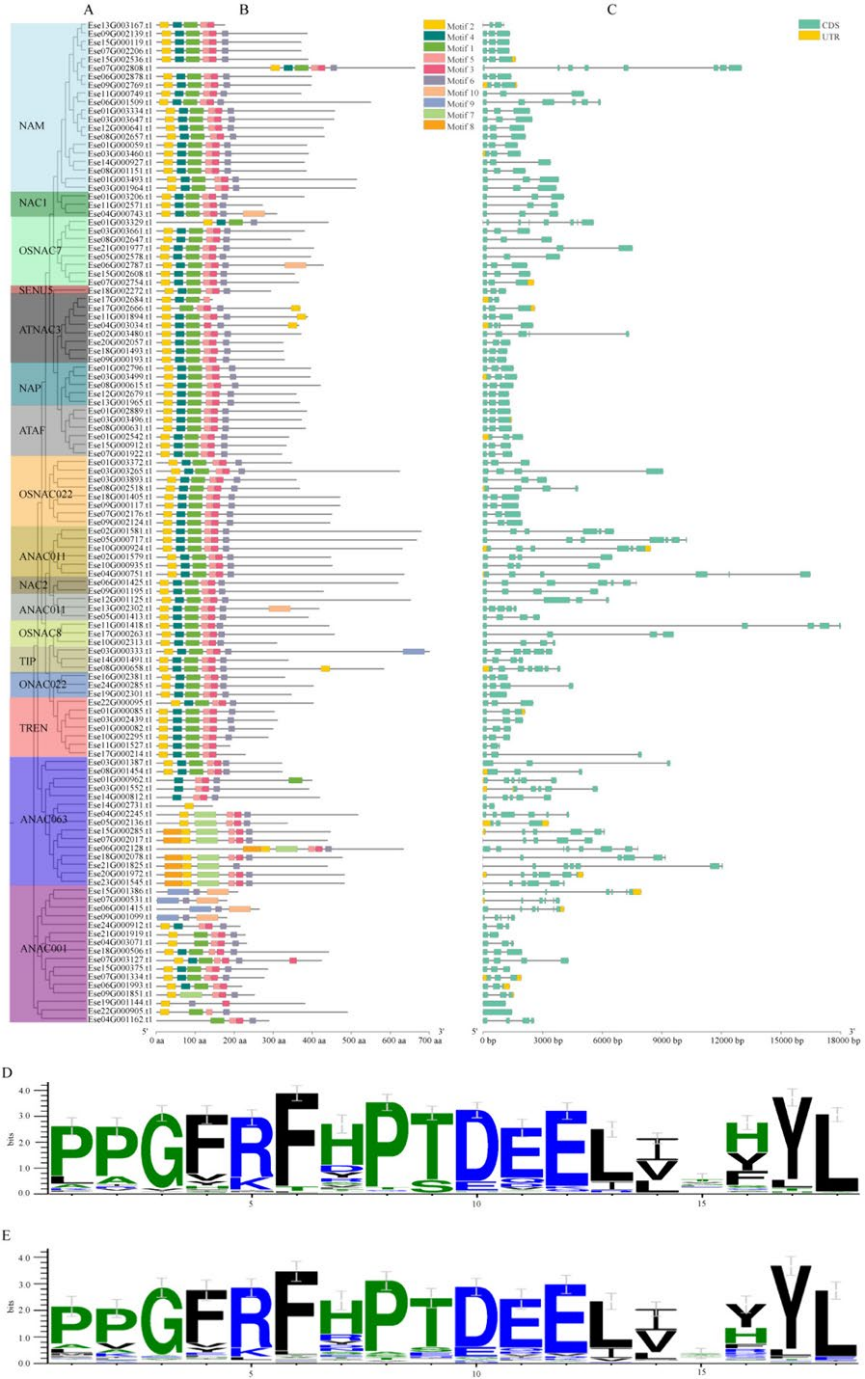
Gene structures, conserved motifs, and core conserved motifs of the NAC gene family in *E. senticosus. ***A** Phylogenetic tree of the *Es*NAC gene family. **B** Distribution of conserved motifs in *Es*NAC genes. **C** Distribution of exons and introns in *Es*NAC genes. Green boxes indicate exons, whereas lines indicate introns. **D** Seqlogo of the core conserved motif 2 in the 117 NAC genes of *E. senticosus*. **E** Seqlogo of the core conserved motif 2 in the 135 NAC genes of *A. thaliana*

A total of 10 conserved motifs (motifs 1–10) were identified in the *Es*NAC gene family (Fig. 3B). Motifs 1, 2, 3, 4, 5, and 6 had the highest frequency. Ese04G000743.t1 and Ese06G002787.t1 contained the highest number of conserved motifs (7 motifs), whereas Ese14G002731.t1 contained only one conserved motif (motif 2). Motif 4 was distributed in all 15 subfamilies (Fig. 3A), motif 9 was found in only the TIP and ANAC001 subfamilies, and motif 8 was unique to the ANAC063 subfamily. These results indicated that the distribution of conserved motifs in *EsNAC* genes exhibited subfamily specificity to a certain extent. Most *Es*NAC TFs contained motif 2 and were distributed in similar locations. In addition, most NAC genes in *A. thaliana* contained motif 2. Therefore, the core conserved motif 2 was constructed for *E. senticosus* (Fig. 3D) and *A. thaliana* (Fig. 3E). The results showed that motif 2 was located near the 5´-amino-terminal 260 aa-318 site in the NAC genes of both species. Therefore, motif 2 was identified as the core conserved structural domain of the NAC gene family. The length of *EsNAC* genes ranged from 150 bp to 1800 bp. *Es*NAC genes contained 1–8 exons, whereas introns were absent in Ese22G000905.t1 and Ese19G001144.t1 in the ANAC001 subfamily. Ese22G000905.t1 contained the longest exon, with all 68 *Es*NAC genes containing 3 exons each, whereas Ese07G002808.t1 contained the largest number of exons (8 exons). Although the distribution of exons and introns greatly differed among the *Es*NAC subfamilies, *Es*NAC genes from the same subfamily usually had a conserved exon–intron distribution pattern. These results indicated that NAC genes in the same subclade had similar structures (Fig. 3 C).

### Analysis of cis-acting elements of *Es*NAC promoters

**Fig. 4:**
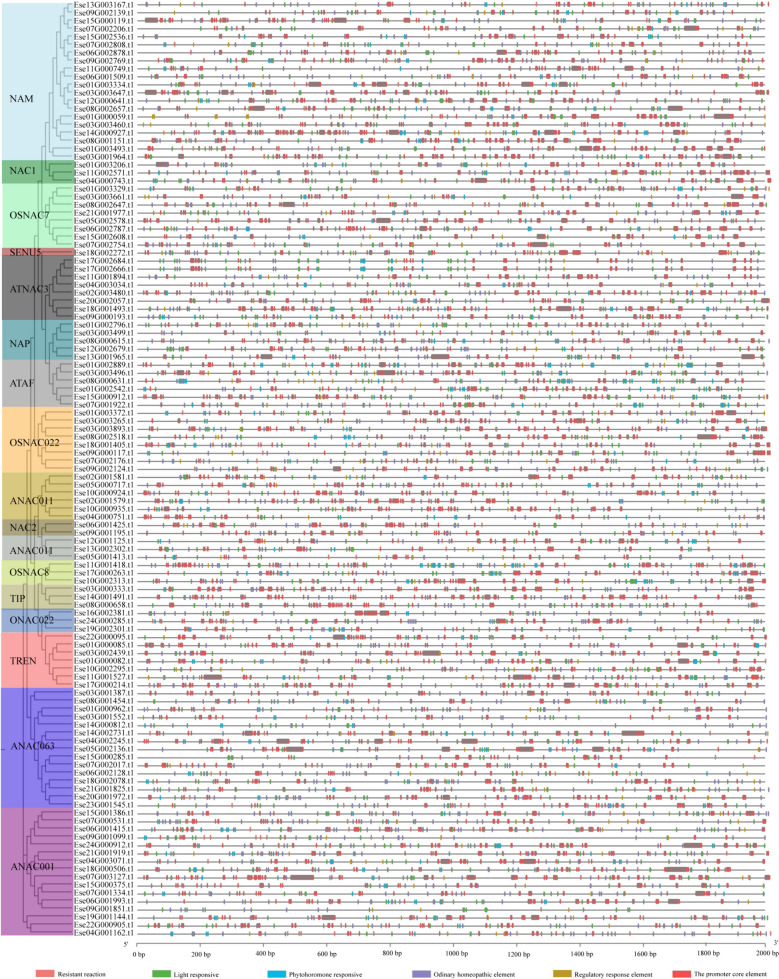
Analysis of cis-acting elements of *EsNAC* promoters

The promoters of the 117 *EsNAC* genes contained six different cis-acting elements (Fig. 4), namely phytohormone response elements, light response elements, resistance response elements, regulatory response elements, promoter core elements (TATA-boxes), and common cis-acting elements (CAAT-boxes). The NAM subfamily contained several cis-acting elements linked to stress response. Among them, five *EsNAC* genes had cis-acting elements related to defense and stress response, while the other five *EsNAC* genes had cis-acting elements associated with response to low-temperature stress. Furthermore, every individual belonging to the *EsNAC* clan possessed at least a single cis-acting component linked to the response of phytohormones.

### Screening of DNA methylation-sensitive *EsNAC* genes

**Fig. 5:**
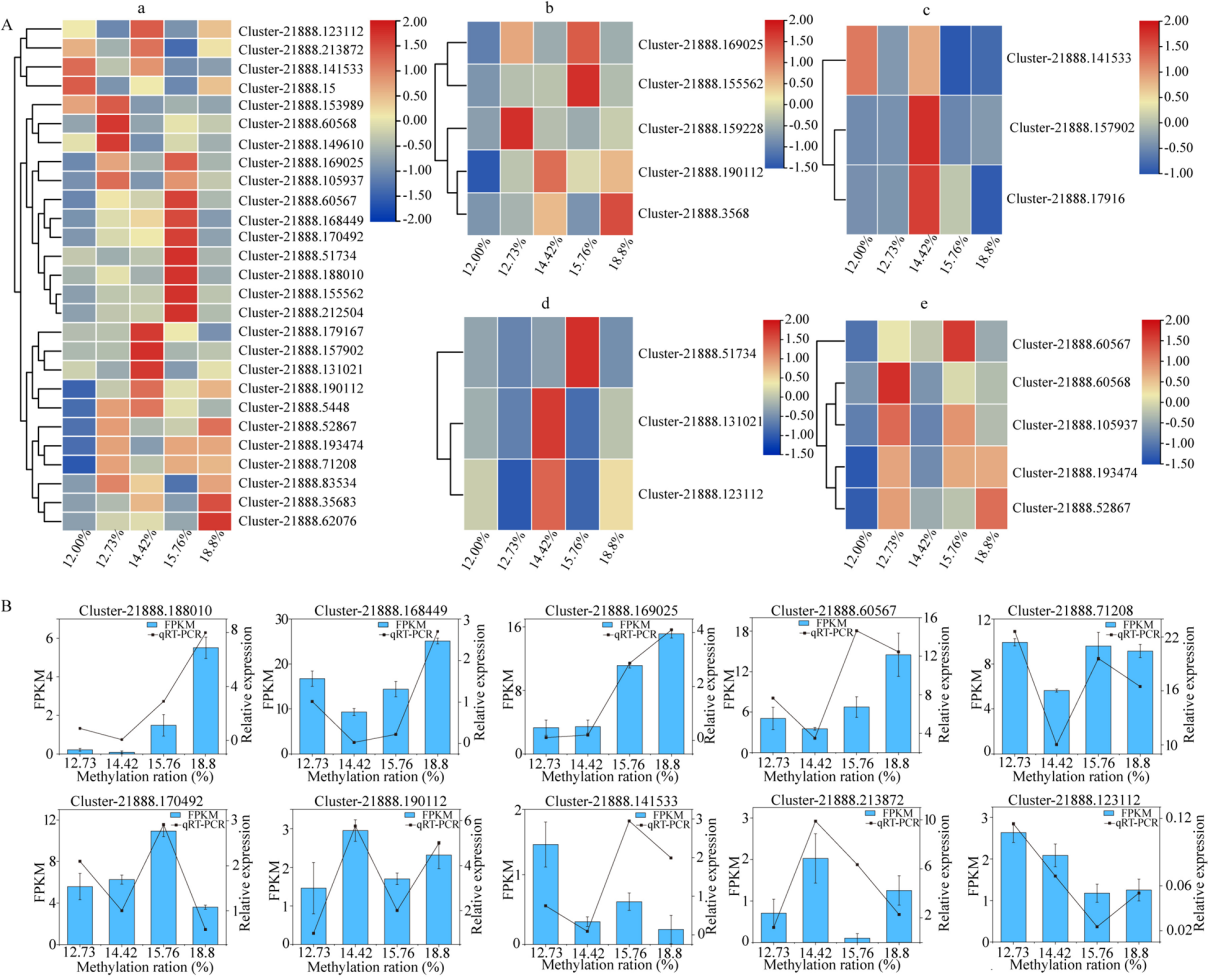
Expression patterns and relative expression of differentially expressed *EsNAC *genes at different methylation rates. **A** Expression of significant *Es*NAC genes at different methylation rates in *E. senticosus*. Note: **a** 27 significantly different *Es*NAC genes; **b** Differentially expressed *Es*NAC genes in the NAM subfamily; **c** Differentially expressed *Es*NAC genes in the NAC1 subfamily; **d** Differentially expressed *Es*NAC genes in the NAP subfamily; **e** Differentially expressed *Es*NAC genes in the ATAF subfamily. **B** Validation of the 10 highly significant differentially expressed *Es*NAC genes via qRT-PCR

The transcriptome sequencing data of *E. senticosus* genes with various DNA methylation levels were compared to the 117 *EsNAC* genes identified in *E. senticosus* using BLAST. A total of 81 *EsNAC* genes were screened for differential expression in different DNA methylation degrees in *E. senticosus*. The expression of 27 *EsNAC* genes was significantly different in different DNA methylation degrees of *E. senticosus* (*P* < 0.05). The relative expression of 27 differentially expressed *EsNAC* genes in different genomic DNA methylation degrees of *E. senticosus* is shown in Fig. 5A. At a 15.76% DNA methylation rate, 8 *EsNAC* genes were upregulated. At an 18.8% methylation rate, 17 *EsNAC* genes were downregulated. The expression patterns of *EsNAC* genes varied among subclades. Most *EsNAC* genes in the ATAF subclade were highly expressed at a 12.73% DNA methylation rate (Fig. 5A-e). Most *EsNAC* genes in the NAM subclade were highly expressed at a 15.76% DNA methylation rate (Fig. 5A-a), whereas no genes were upregulated at a 12.00% methylation rate. In addition, large differences in the expression patterns of different genes were observed in the same subfamily.

In order to confirm the dependability of the transcriptome sequencing data of *E. senticosus*, a total of 10 *EsNAC* genes exhibiting remarkably different expression (*P* < 0.01) were identified using qRT-PCR. The results showed that the relative expression of *EsNAC* genes was consistent with the transcriptome sequencing data (Fig. 5B). These results indicated that *EsNAC* genes associated with DNA methylation, that is, *EsNAC* genes that are sensitive to DNA methylation, could be identified using the transcriptome sequencing data of DNA methylation-associated genes of *E. senticosus*.

Correlation analysis revealed that Cluster-21888.71208 (named *EsJUB1*), Cluster-21888.170492 (named *EsNAC047*), Cluster-21888.190112 (named *EsNAC098*), and Cluster-21888.213872 (named *EsNAC005*), which belong to the *Es*NAC transcription factor family, exhibited the strongest correlation with DNA methylation ratio. In particular, the expression of *EsNAC098* and *EsNAC005* exhibited a positive correlation with DNA methylation (Pearson correlation coefficients; *P* = 0.969491 and *P* = 0.481879), whereas that of *EsJUB1* and *EsNAC047* exhibited a negative correlation with DNA methylation (Pearson correlation coefficients; *P* = -0.99831 and *P* = -0.71914).

### Subcellular localization of *Es*NAC proteins

**Fig. 6:**
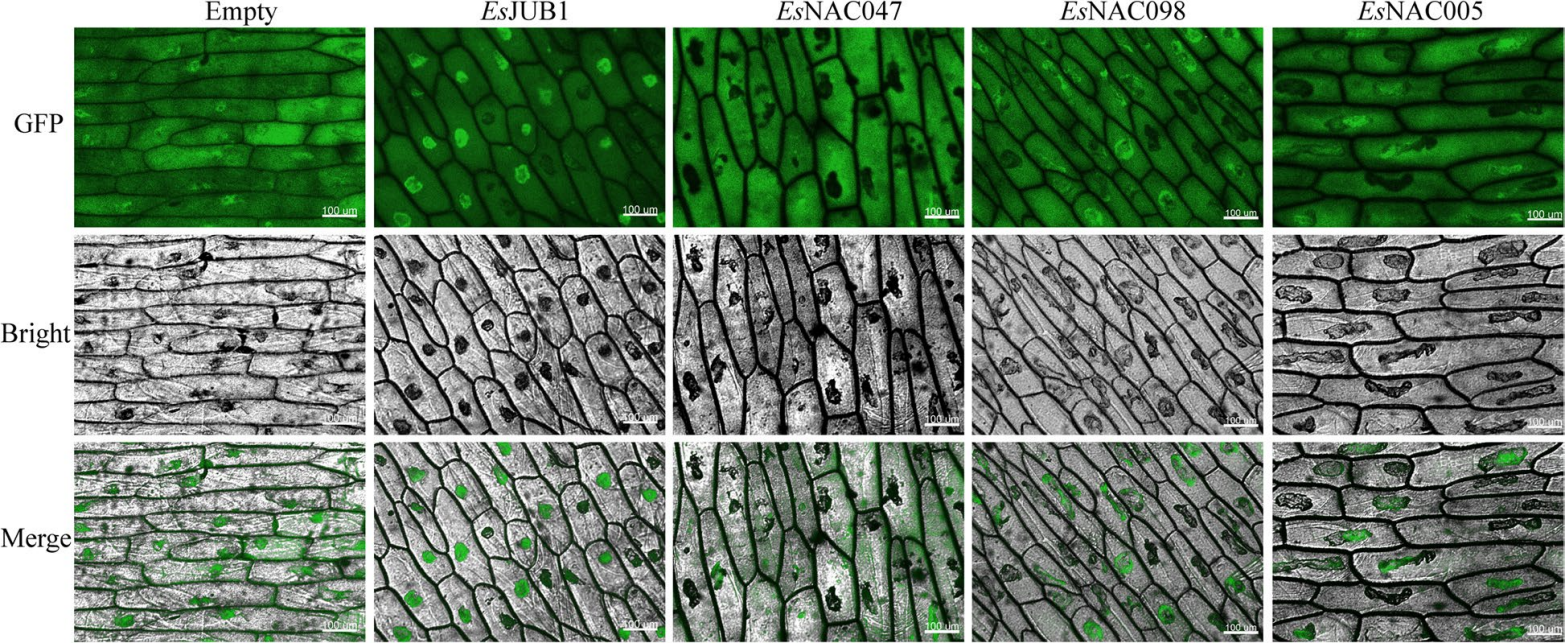
Subcellular localization of *Es*NAC proteins

According to the predictions of WoLF PSORT, *Es*JUB1 and *Es*NAC005 were found in the nucleus, while *Es*NAC047 and *Es*NAC098 were found in both the nucleus and cytoplasm. In order to confirm the precision of these forecasts, the four *Es*NAC transcription factors were cloned in expression vectors containing GFP and briefly expressed in the epidermal cells of onions. The GFP signals of *Es*JUB1 and *Es*NAC005 were detected in the nucleus, whereas those of *Es*NAC047 and *Es*NAC098 were detected in the cytoplasm and nucleus (Fig. 6). These results were consistent with the predicted locations of the four TFs in WoLF PSORT.

### Effects of DNA methylation on the activities of *EsFPS*,*EsSS*, and *EsSE* promoters

**Fig. 7:**
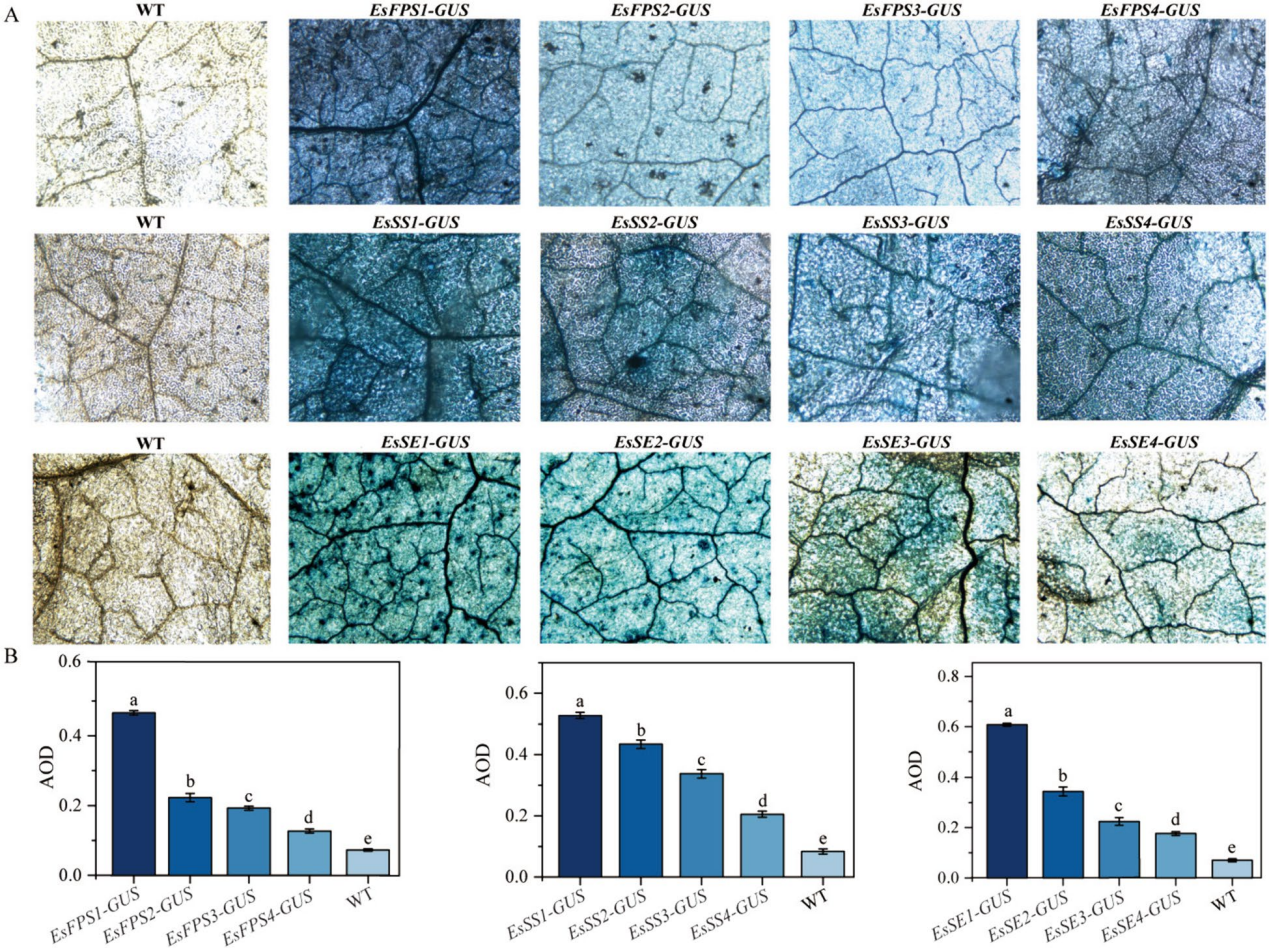
Effects of DNA methylation on the activities of *EsFPS*, *EsSS*, and *EsSE *promoters. **A** GUS staining of *N. benthamiana* transfected with *EsFPS*, *EsSS*, and *EsSE* promoters with different DNA methylation degrees (magnification: 4×). **B** Average optical density of GUS-stained leaves. a, b, c, d, and e represent significance (*P* < 0.05). WT: wild type; *EsFPS1*, *EsSS1*, and *EsSE1-GUS1*: unmethylated *EsFPS*, *EsSS*, and *EsSE* promoters; *EsFPS2*, *EsSS2*, and *EsSE2-GUS*: *EsFPS*, *EsSS*, and *EsSE* promoters methylated for 20 min; *EsFPS3*, *EsSS3*, and *EsSE3- GUS*: *EsFPS*, *EsSS*, and *EsSE* promoters methylated for 40 min; *EsFPS4*, *EsSS4*, and *EsSE4-GUS*: *EsFPS*, *EsSS*, and *EsSE* promoters methylated for 60 min

The GUS staining results indicated that *Nicotiana benthamiana* leaves, except for wild-type ones, were all stained blue, demonstrating that the GUS gene expression could be activated by the *EsFPS*, *EsSS*, and *EsSE* promoters regardless of DNA methylation (Fig. 7A). The promoters of *EsFPS*, *EsSS*, and *EsSE* genes without DNA methylation exhibited the highest activity and the darkest blue color in transgenic *N. benthamiana* leaves. The staining intensity of *N. benthamiana* leaves decreased significantly (*P* < 0.05) with an increase in the duration of catalytic action of DNA methyltransferase. These results indicated that the activity of *EsFPS*, *EsSS*, and *EsSE* promoters decreased significantly with an increase in their DNA methylation degree. Altogether, the degree of inhibition of *EsFPS* and *EsSE* promoter activity by DNA methylation was significantly higher than that of the *EsSS* promoter activity (Fig. 7B).

### Analysis of the transcriptional activation activity of *EsNAC* genes

**Fig. 8:**
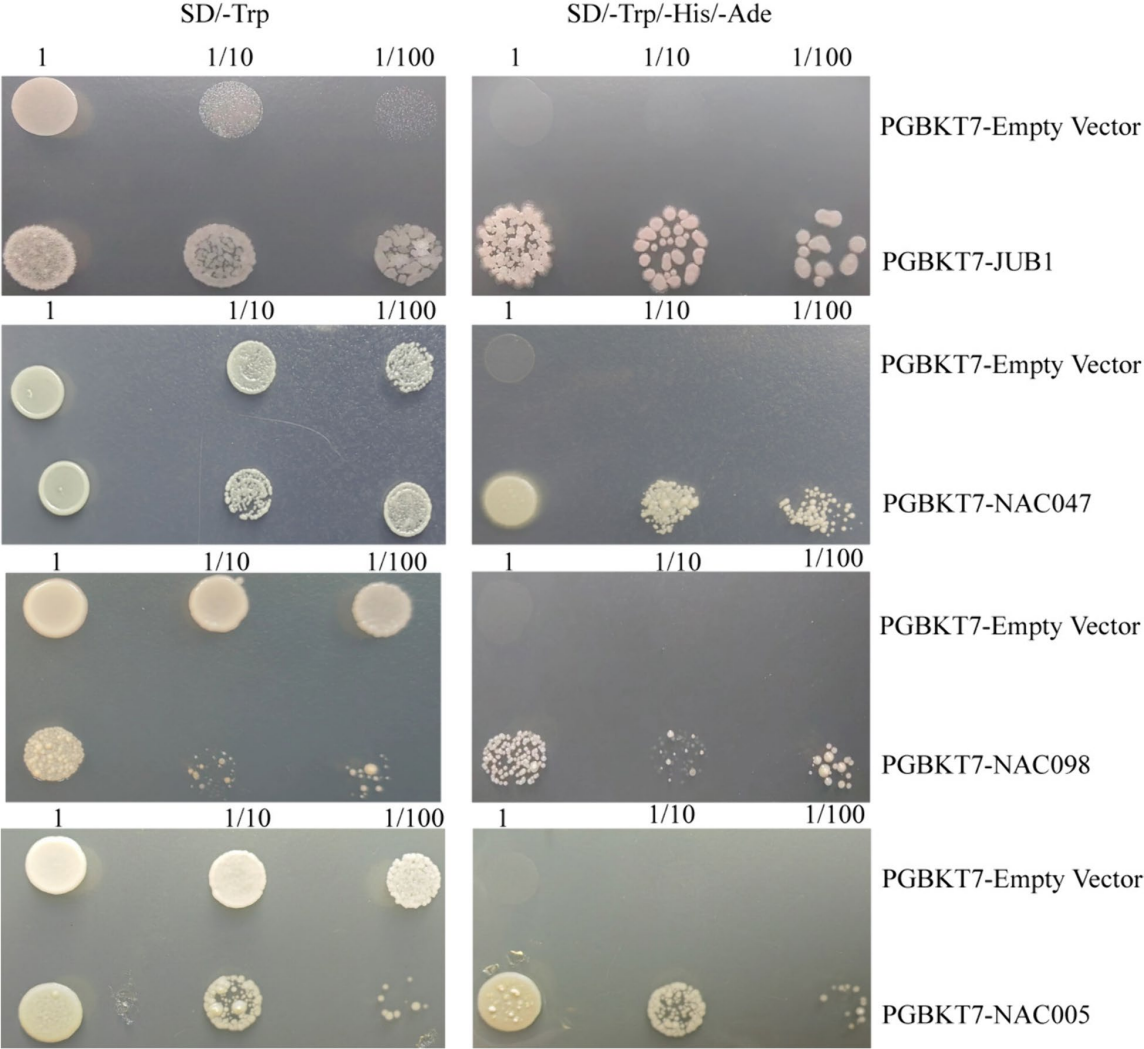
Yeast mono-hybridization experiments of *Es*NAC

Note: SD/-Trp, tryptophan-deficient medium; SD/-Trp/-His/Ade, Tryptophan/histidine/adenine-deficient medium. 1, undiluted bacterial solution; 1/10, 10-fold diluted bacterial solution; 1/100, 100-fold diluted bacterial solution.

The outcomes of yeast mono-hybridization indicated that the yeast strains transfected with either the PGBKT7-*Es*JUB1, -*Es*NAC047, -*Es*NAC098, or -*Es*NAC005 fusion vector or the PGBKT7 empty vector exhibited normal growth on the SD/-Trp medium (Fig. 8), suggesting the successful transfection of exogenous plasmids into AH109 yeast cells. The yeast strains transfected with the PGBKT7-NAC fusion vector exhibited normal growth on both SD/-Trp and SD/-Trp/-His/-Ade media; in contrast, the yeast strains transfected with the pGBKT7 empty vector only showed normal growth on the SD/-Trp medium. The number of normally growing colonies showed a decreasing trend at 1, 1/10, and 1/100 dilutions. These results indicated that *Es*JUB1, *Es*NAC047, *Es*NAC098, and *Es*NAC005 TFs could bind to promoters and activate the expression of downstream target genes in yeast cells, thus exhibiting strong transcriptional activation activity.

### Effects of DNA methylation on the binding of *Es*NAC to *EsFPS*,*EsSS, * and *EsSE* promoters

**Fig. 9:**
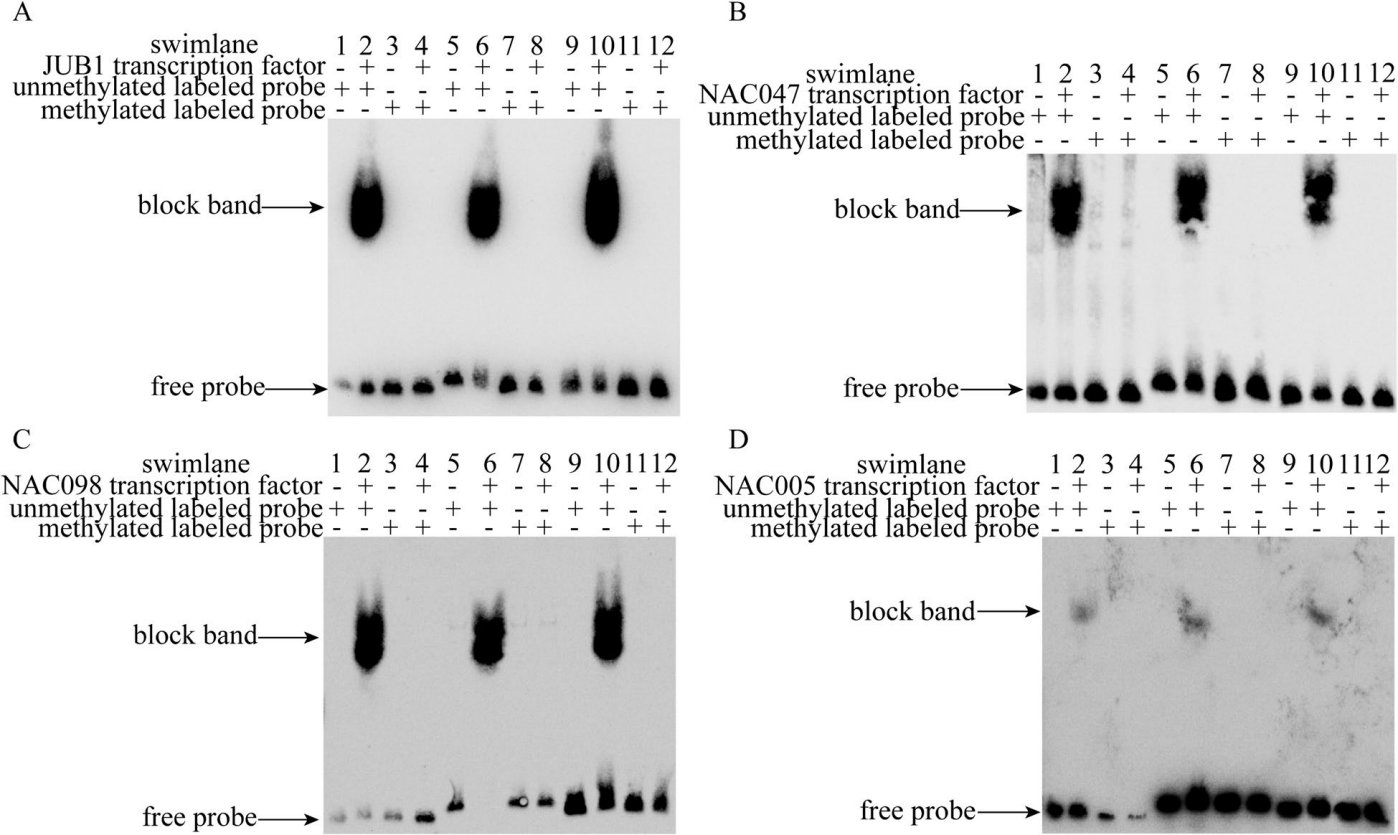
EMSA of *EsFPS*, *EsSS*, and *EsSE *promoters and *EsNAC *genes. **A** Binding of *Es*JUB1 to *EsFPS*, *EsSS*, and *EsSE* probes. **B** Binding of *Es*NAC047 to *EsFPS*, *EsSS*, and *EsSE* probes; **C** Binding of *Es*NAC098 to *EsFPS*, *EsSS*, and *EsSE* probes. **D** Binding of *Es*NAC005 to *EsFPS* , *EsSS* , and *EsSE* probes. Note: “+” indicates the inclusion of the component, whereas “-” indicates its exclusion

TFPred was used to predict whether *Es*NAC could bind to methylated *EsFPS*, *EsSS*, and *EsSE* promoters (Supplementary Table 3), and JASPAR was used to predict the binding sites of *Es*JUB1, *Es*NAC047, *Es*NAC098, and *Es*NAC005 to *EsFPS*, *EsSS*, and *EsSE* promoters. In addition, the location of CpG islands in *EsFPS*, *EsSS*, and *EsSE* promoters was analyzed. The binding sites and CpG island sites were intersected to obtain probe sequences for EMSA (Supplementary Table 3). *Es*JUB1, *Es*NAC047, *Es*NAC098, and *Es*NAC005 expressed and purified in vitro were subjected to EMSA with biotin-labeled probes of *EsFPS*, *EsSS*, and *EsSE* promoters. The results showed that *Es*JUB1, *Es*NAC047, *Es*NAC098, and *Es*NAC005 could bind to unmethylated *EsFPS*, *EsSS*, and *EsSE* promoter probes to form complexes with larger molecular mass, resulting in a slower migration in the gel and the formation of pronounced lagging bands in the lane. On the contrary, methylated *EsFPS*, *EsSS*, and *EsSE* promoter probes could not form complexes with *Es*JUB1, *Es*NAC047, *Es*NAC098, and *Es*NAC005 (Fig. 9). These results indicate that *Es*JUB1, *Es*NAC047, *Es*NAC098, and *Es*NAC005 cannot bind to methylated DNA, that is, DNA methylation inhibits the binding of *Es*JUB1, *Es*NAC047, *Es*NAC098, and *Es*NAC005 to target DNA.

### Molecular dynamics of DNA methylation-induced inhibition of the binding of *Es*NAC to *EsFPS*, *EsSS*, and *EsSE* promoters

**Fig. 10:**
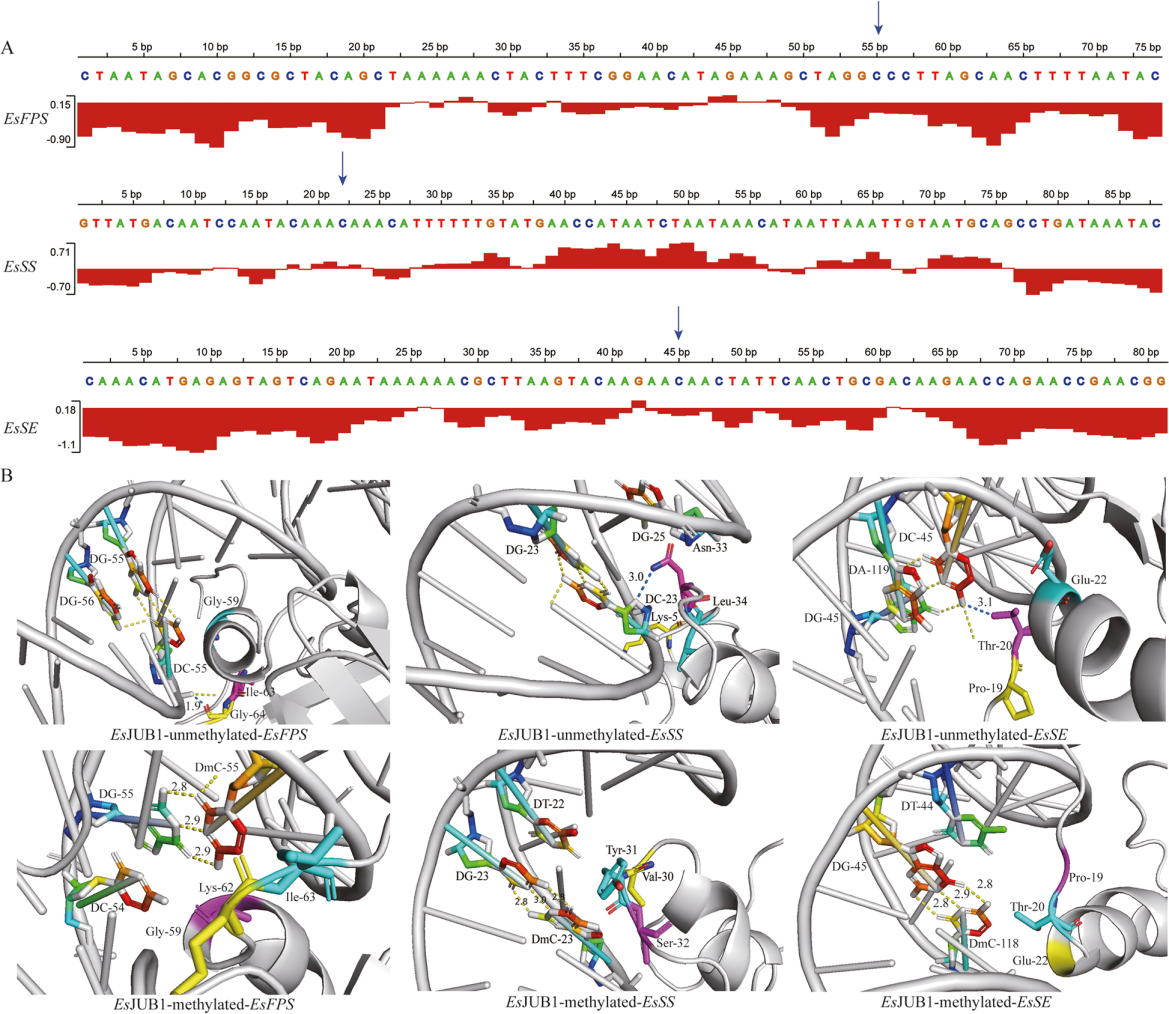
Rigidity analysis of *EsFPS*, *EsSS*, and *EsSE* promoters and their molecular docking with *Es*JUB1. **A** Rigidity analysis of *EsFPS*, *EsSS*, and *EsSE* promoters; the positions indicated by arrows are the binding sites of *Es*JUB1. **B** Molecular docking of *Es*JUB1 with *EsFPS*, *EsSS*, and *EsSE* promoters at different methylation degrees. Note: Blue dotted lines represent the docked hydrogen bonds of the binding sites, yellow dotted lines represent the undocked hydrogen bonds, and the number next to the hydrogen bond represents the bond length

The average rigidity of the *EsFPS* promoter probe (-0.327200689) was 1.62 times higher than that of the binding sites of *Es*JUB1, *Es*NAC047, *Es*NAC098, and *Es*NAC005 (-0.20140678). The average rigidity of the *EsSS* promoter probe (0.040219949) was 0.35 times higher than that of the binding sites (0.116152555). The average rigidity of the *EsSE* promoter probe (-0.468000915) was 2.42 times higher than that of the binding sites (-0.19357401). These results suggest that the curvature of cytosine in TFBSs is significantly higher than that in other regions, which may favor the binding of *Es*JUB1, *Es*NAC047, *Es*NAC098, and *Es*NAC005 to target DNA (Fig. 10A).

The results of molecular docking showed that *Es*JUB1 (Fig. 10B), *Es*NAC047, *Es*NAC098, and *Es*NAC005 (Supplementary Fig. 1) could successfully bind to unmethylated *EsFPS*, *EsSS*, and *EsSE* promoters. Unmethylated cytosine DC-55 of the *EsFPS* promoter, DC-23 of the *EsSS* promoter, and DC-45 of the *EsSE* promoter formed hydrogen bonds with specific amino acids in the grooves or pockets present in *Es*JUB1, *Es*NAC047, *Es*NAC098, and *Es*NAC005 to achieve the binding of TFs to target DNA. On the contrary, DNA methylation at the abovementioned sites increased their distance from specific amino acids and prevented the formation of hydrogen bonds, thereby blocking the binding of *Es*JUB1 (Fig. 10B), *Es*NAC047, *Es*NAC098, and *Es*NAC005 (Supplementary Fig. 1) to methylated *EsFPS*, *EsSS*, and *EsSE* promoters.

### Effects of *Es*NAC binding on the activities of *EsFPS*, *EsSS*, and *EsSE* promoters

**Fig. 11:**
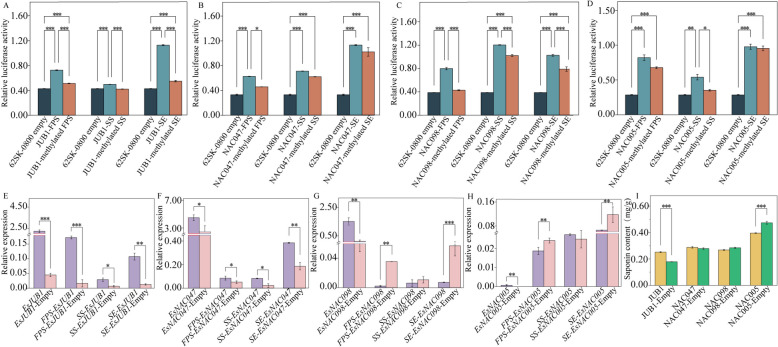
Dual-luciferase reporter assay of *Es*NAC with *EsFPS*, *EsSS*, and *EsSE* promoters and the effects of overexpression of *Es*NAC on the synthesis of *E. senticosus* saponins. **A** Activity of *Es*JUB1 after binding to *EsFPS*, *EsSS*, and *EsSE* promoters before and after methylation. **B** Activity of *Es*NAC047 after binding to *EsFPS*, *EsSS*, and *EsSE* promoters before and after methylation. **C** Activity of *Es*NAC098 after binding to *EsFPS*, *EsSS*, and *EsSE* promoters before and after methylation. **D** Activity of *Es*NAC005 after binding to *EsFPS*, *EsSS*, and *EsSE* promoters before and after methylation. **E** Changes in the expression of *EsFPS*, *EsSS*, and *EsSE* after overexpression of *Es*JUB1. **F** Changes in the expression of *EsFPS*, *EsSS*, and *EsSE* after overexpression of *Es*NAC047. **G** Changes in the expression of *EsFPS*, *EsSS*, and *EsSE* after overexpression of *Es*NAC098. **H** Changes in the expression of *EsFPS*, *EsSS*, and *EsSE* after overexpression of *Es*NAC005. **I** Changes in the content of *E. senticosus* saponins after overexpression of *Es*NAC. Note: *, *P* < 0.05; **, *P* < 0.01; ***, *P* < 0.001

Dual-luciferase reporter assay showed that the relative luciferase activity of all samples was significantly higher than that of control samples, indicating that the construction of vectors was successful (Fig. 11A–D). The activity of methylated *EsFPS*, *EsSS*, and *EsSE* promoters was reduced to different degrees after their binding to *Es*JUB1, *Es*NAC047, *Es*NAC098, and *Es*NAC005. The activity of methylated *EsSE* promoter bound to *Es*NAC047 and *Es*NAC005 did not reduce significantly (*P* < 0.001). On the contrary, the activity of methylated *EsFPS* promoter bound to *Es*JUB1 exhibited the highest reduction of 51.42%. These results indicated that DNA methylation in *EsFPS*, *EsSS*, and *EsSE* promoters hindered the binding of *Es*JUB1, *Es*NAC047, *Es*NAC098, and *Es*NAC005 to the promoters and reduced the initiation efficiency of the promoters.

### Overexpression analysis of *EsNAC* genes

qRT-PCR revealed that the expression of *EsJUB1*, *EsNAC047*, *EsNAC098*, and *EsNAC005* was significantly higher in the overexpression group than in the control (empty vector) group (Fig. 11E–H) (*P* < 0.05), indicating that overexpression of the genes was successfully induced. After the overexpression of *EsJUB1* and *EsNAC047*, the expression of *EsFPS*, *EsSS*, and *EsSE* genes was significantly elevated (*P* < 0.05). After the overexpression of *EsNAC098* and *EsNAC005*, the expression of *EsFPS*, *EsSS*, and *EsSE* genes was reduced to different degrees. The expression of *EsFPS* was most elevated (90.21%) after the overexpression of *EsJUB1*, whereas the expression of *EsSE* was most reduced (87.23%) after the overexpression of *EsNAC098*. Furthermore, the total saponin content was increased after the overexpression of *EsJUB1* and *EsNAC047* and decreased after the overexpression of *EsNAC098* and *EsNAC005* (Fig. 11I). Overexpression of *EsJUB1* increased the total saponin content to 129.05% of the control, whereas overexpression of *EsNAC005* decreased the total saponin content by 16.44%.

## Discussion

Sequencing has been performed on the genomes of *Panax ginseng* [27], *Panax notoginseng* [28], *A. elata* [29], and various other medicinal plants belonging to the Araliaceae family. In their study, Yang and colleagues [7] conducted chromosome-scale sequencing and assembly of *E. senticosus* genome, revealing a total of 36,372 coding genes in the genome with a chromosome count of 2n = 48. In addition, a large number of functional genes and TFs adapted to growth in cold regions were retained and expanded. The duplication of specific genes, chromosomes, and entire genomes is significant in shaping the structure of the plant genome and the evolution of genes [30]. In this study, 117 *NAC* genes were identified in the genome of *E. senticosus*. Most of these genes were closely arranged on chromosomes as gene clusters and contained multiple pairs of tandemly duplicated genes. Further analysis of the intra- and inter-chromosomal colinearity of *EsNAC* genes revealed a large number of *NAC* covariates. In addition, significant colinearity of *NAC* genes was found in *A. elata*, which is a member of the Araliaceae family. These findings indicate that *NAC* genes evolve through tandem duplication and chromosome segment duplication to form interspecies-specific genes in Araliaceae [29]. *Eucommia ulmoides* [31], *Saccharum officinarum* [32], and *Kandelia obovate* [33] have also exhibited comparable instances of gene duplication.

Different plants exhibit variations in the quantity of NAC gene family members and the categorization of the clades. *Es*NAC was categorized into 15 subfamilies based on the classification of NAC proteins in *A. thaliana*. The number of *Es*NAC subfamilies resembled *Isatis tinctoria* with 16 subfamilies [23], surpassing *Solanum lycopersicum* with 12 subfamilies [34], *Scutellaria baicalensis* with 6 subfamilies [35], and *Juglans regia* with 8 subfamilies [36]. Additionally, the count of NAC gene family members varied among these species [31]. The considerable diversity in *NAC* genes across plant species implies that despite their shared ancestry, different species exhibit distinct evolutionary patterns. Therefore, some *NAC* genes may have been lost or expanded during evolution.

Conserved structural sequences are a prerequisite for conserved biological functions. Studies have shown that plant NAC gene families usually contain a specific highly conserved N-terminal structural domain [31]. In this study, the conserved motifs of *EsNAC* genes in the same subfamily were found to be similar, and a highly conserved motif 2 (“PPGFRFHPTDEELIIHYL” motif) distributed at the 5´-amino-terminal end, was identified in the *NAC* genes of both *E. senticosus* and *A. thaliana*. These findings are consistent with those of a study on Chinese cabbage [37]. UTR is closely associated with gene expression motifs that enhance plant perception of developmental and environmental regulatory factor stimuli [38]. For example, drought stress induces the elongation of 3’-UTR transcripts, which can lead to the acquisition of novel functions [39]. The length of UTRs varies among the members of the *EsNAC* gene family, which indicates that different *EsNAC* genes may have different functions and play different roles in different environments.

NAC TFs play an important role in plant response to stress [40]. As important molecular switches, cis-acting elements contribute to the transcriptional regulation of dynamic gene networks in response to different abiotic stresses [41]. The promoters of *EsNAC* genes were found to contain a large number of elements involved in defense and response to stress conditions such as low temperature and drought. This finding indicates that the *NAC* gene family may be involved in life activities such as response to abiotic stresses in *E. senticosus*.

Drought-induced elevation of DNA methylation levels may represent one of the major mechanisms through which plants cope with drought stress [42]. During this investigation, the manifestation of certain *EsNAC* genes displayed comparable patterns of upregulation or downregulation across varying levels of DNA methylation. Nevertheless, Cluster-21888.60567, Cluster-21888.60568, and Cluster-21888.52867, members of the ATAF subfamily, exhibited distinct expression profiles, indicating their participation in the comprehensive control of diverse DNA methylation reactions. Studies have validated the role of ATAF subfamily members in response to various biotic or abiotic stresses [43]. The Pearson correlation analysis revealed a significant association between DNA methylation in *E. senticosus* and the expression of *Es*JUB1, *Es*NAC047, *Es*NAC098, and *Es*NAC005. The discovery implies that the four *Es*NAC genes have a significant function in facilitating the regulatory impacts of DNA methylation on secondary metabolism in *E. senticosus*.

In previous research, it has been demonstrated that the ONAC045 protein [44], which enhances the ability of rice to withstand drought and tolerate salt, and the SbSNAC1 protein [45], which grants drought tolerance in transgenic *A. thaliana*, are both situated within the nucleus. All *EsNAC* genes in this study were observed to be situated in either the nucleus or cytoplasm, aligning with the outcomes of prior investigations. The whole sequence of *EsNAC* exhibits strong transcriptional self-activation activity, which can induce the expression of downstream target genes. The sequence of *EsNAC* is different from that of *CaNAC064* in peppers, which lacks transcriptional self-activation activity owing to the presence of a transcription-repressive region at 1–690 bp [46]. According to prior research, an overabundance of DNA methylation has been found to result in the suppression of gene transcription [47]. Consistently, this study showed that the promoter initiation efficiency gradually decreased with an increasing DNA methylation level of *EsFPS*, *EsSS*, and *EsSE* promoters.

Methylation of TFBSs was previously thought to either inhibit the binding of methylation-sensitive TFs or have no effect on the binding of methylation-insensitive TFs [48]. However, recent studies employing high-throughput analysis have reported that methylation within TFBSs can lead to either a decrease or an increase in TF binding based on the location of the methylation site within TFBSs. In addition, this location determines the strength of the effect of methylated TFBSs on TF binding affinity [49]. Based on differences in the binding force response of human TFs to methylated TFBSs, DNA methylation can be divided into three types as follows: DNA methylation that inhibits TF binding, promotes TF binding, and does not affect TF binding. Methylation-sensitive transcription factors, encompassing the initial two categories, have a significant function in governing gene expression through DNA methylation. In a previous study, the results of DNA affinity purification sequencing showed that 72% of TF binding in *A. thaliana* was inhibited by DNA methylation at TFBSs, 4% were promoted, and 24% were unaffected or weakly affected [50]. In this study, the results of in vitro validation of *Es*JUB1, *Es*NAC047, *Es*NAC098, and *Es*NAC005 showed that methylation of the promoter-binding site in key enzyme-coding genes inhibited the binding of *Es*NAC to the promoters.

DNA rigidity is closely related to gene transcription, enhanced regulation, and TF binding and is mainly involved in transcriptional regulation in terms of DNA mechanical properties [51]. Unmethylated DNA has lower rigidity, whereas methylated DNA has higher rigidity [52]. In this study, assessment of the rigidity of *EsFPS, EsSS*, and *EsSE* promoters of *E. senticosus* showed that rigidity at the *EsNAC*-binding sites was less than the average rigidity of the promoters. This finding suggests that DNA methylation increases DNA rigidity at the *Es*NAC-binding site, which may prevent the binding of *Es*NAC to methylated DNA. Furthermore, we performed molecular docking of *EsFPS, EsSS*, and *EsSE* promoters with specific structural domains of *Es*NAC to elucidate the molecular mechanism of nucleotides and amino acids at the binding site. Unlike the ORE1-NAC homodimer that mediates binding to target DNA sequences through extensive electrostatic interactions and the conserved residues Lys102 and Thr104 [21], *Es*NAC generated binding pockets or grooves at the binding site and subsequently interacted with unmethylated DNA through hydrogen bonding. Methylated cytosine alters the 3D structure of target DNA [53], the hydrophobic methyl groups in methylated cytosine directly block the binding between the *Es*NAC groove and target DNA, forming a physical barrier [54, 55]. Consequently, the distance between the methylated DNA and *Es*NAC amino acid residues increases, preventing the generation of hydrogen bonds and resulting in the failure of docking. However, specific amino acids required for the binding of *Es*NAC to target DNA were not identified in this study.

Studies on banana have shown that *Ma*NAC1 mediates the response of the secondary wall to low temperature by binding to *Ma*CESA6B and *Ma*CESA7 promoters and directly activating their transcription [56]. The study discovered that *Es*NAC effectively binds to and stimulates the promoters of crucial enzyme-coding genes related to saponin synthesis, specifically *EsFPS*, *EsSS*, and *EsSE*, consequently initiating their transcription. However, the activation activities of *Es*JUB1, *Es*NAC047, *Es*NAC098, and *Es*NAC005 were significantly reduced with an increase in the methylation levels of the promoters. These results indicate that DNA methylation inhibits the transcriptional activation activity of *Es*NAC bound to *EsFPS*, *EsSS*, and *EsSE* promoters.

Earlier research has indicated a strong correlation between the expression of a specific group of *PgNAC* genes and the key enzyme-coding genes related to the synthesis of ginsenoside and saponin in *P. ginseng*. In addition, overexpression of *PgNAC42-1* promotes saponin biosynthesis [57]. In tomato, the NAC TF NOR can directly positively regulate the expression of senescence-related genes and lead to progressive plant senescence [58]. Overexpression of *Es*JUB1 and *Es*NAC047, which are negatively associated with DNA methylation, upregulated the expression of *EsFPS*, *EsSS*, and *EsSE*, resulting in increased synthesis of saponins. On the contrary, overexpression of *Es*NAC098 and *Es*NAC005, which are positively associated with DNA methylation, decreased the expression of *EsFPS*, *EsSS*, and *EsSE*, resulting in decreased synthesis of saponins. These findings are consistent with those of a previous study on *E. senticosus* [8, 10] and demonstrate the functional diversity of *Es*NAC gene family members. The overabundance of *Es*NAC047 and *Es*NAC098 had no notable impact on the expression of *EsFPS*, *EsSS*, and *EsSE*, indicating that additional regulators might be implicated in the synthesis of saponins in *E. senticosus*.

The establishment of linkage networks between TFs and DNA methylation can offer valuable insights for plant breeding and crop enhancement [59]. A study on *Camellia sinensis* identified 27 transcription factors, including NAC, that exhibited significant correlations with gene expression in metabolic pathways through co-expression analysis. Methylated DNA was found to impact the binding of NAC transcription factors, thereby regulating the expression of associated genes and influencing the metabolism of flavonoids, theanine, and caffeine [60]. Research by Cui et al. [10] demonstrated that alterations in DNA methylation of the *E. senticosus* MDD promoter, under the influence of DNA methyltransferase, could modulate its expression by affecting the binding affinity of transcription factors. This, in turn, could impact the expression of other genes in the terpenoid synthesis pathway and the production of saponins in *E. senticosus*. The study further revealed that DNA methylation of *EsFPS*, *EsSS*, and *EsSE* promoters in *E. senticosus* regulates the expression of related genes by influencing the binding capacity of NAC transcription factors, ultimately governing the biosynthesis of secondary metabolites such as saponins. Thus, the investigation elucidated the molecular mechanism by which DNA methylation mediated by NAC in *E. senticosus* regulates saponin synthesis.

## Conclusion

A total of 117 *EsNAC* genes were discovered in *E. senticosus* during this investigation, revealing that they replicate through both tandem duplication and chromosome segment duplication. Motif 2 was identified as the core conserved motif of *EsNAC*. The cis-acting elements, gene structures, and expression patterns of each *EsNAC* gene were different. *Es*JUB1, *Es*NAC047, *Es*NAC098, and *Es*NAC005 were localized in the nucleus or cytoplasm and exhibited transcriptional self-activation activity. DNA methylation significantly reduced the activity of the *EsFPS*, *EsSS*, and *EsSE* promoters and inhibited the binding of *Es*JUB1, *Es*NAC047, *Es*NAC098, and *Es*NAC005 to these promoters. These changes further altered the expression of *EsFPS*, *EsSS*, and *EsSE*, eventually altering saponin synthesis in *E. senticosus*. These results find that *EsNAC* plays a significiant function in mediating the regulatory impacts of DNA methylation on saponins synthesis in *E. senticosus*. This study may help to investigate the function of *EsNAC* genes and contribute to the molecular breeding of high-quality *E. senticosus*.

## Materials and methods

### Source of data and screening of *EsNAC* genes

The hidden Markov model of *NAC* genes (PF01849, PF02365) was downloaded from the PFAM database (http://pfam.xfam.org/). Using the HMMER program, the genomic data of *E. senticosus* was searched to find the NAC protein domain, with an E-value of 0.001 [7]. To build a domain model specific to the species, BLAST was utilized, with the E-value adjusted to 0.001. The sequence obtained from the second search was sent to the NCBI CD-Search tool SMART (http://smart.embl-heidelberg.de/smart/set_mode.cgi? NORMAL = 1). Eventually, *Es*NAC family genes were confirmed.

### Chromosome localization and collinearity of *EsNAC* genes

The mapping of *EsNAC* genes on chromosomes was done using Mapchart (https//www.wur.nl/en/show/Mapchart.htm). To establish the collinearity relationship of *Es*NAC protein amino acid sequences within species, we utilized MCScanX [61] available in TBtools (https//github.com/CJ-Chen/TBtools). The *NAC* genes from *A. elata* [29] were screened and identified. Additionally, an analysis was conducted on the *NAC* genes of *E. senticosus* and *A. elata* to evaluate the collinear relationship between different species [62].

### Construction and classification of *Es*NAC phylogenetic tree

The protein sequence of *NAC* in *A.thaliana* was acquired from the TAIR database (https//www.arabidopsis.org/). The PFAM and SMART tools provided by NCBI CD-Search (http://smart.embl-heidelberg.de/smart/set_mode) can be utilized. A total of 135 *A. thaliana NAC* genes were screened using NORMAL = 1, and 16 genes from various subgroups were chosen. The sequences of the 16 NAC proteins were used as an outgroup for multiple sequence comparisons of the 117 *Es*NAC proteins of *E. senticosus* using ClstalW. MEGAX software was used to construct a neighbor-joining (NJ) phylogenetic tree, with a bootstrap value of 1000. Based on the classification method and phylogenetic relationships described in a study conducted [22], the *EsNAC* genes were categorized into clades.

### Analysis of the conserved motifs, gene structures, and promoters of *EsNAC *genes

The prediction of the conserved motifs of *EsNAC* genes was conducted using MEME (http://meme-suite.org). The parameters were set as follows: the total number of motifs searched was 10, with a minimum length of 6 and a maximum length of 50. The conserved motifs and structures of introns and exons were visualized using TBtools [61]. Weblogo (https //weblogo.berkeley.edu/logo.cgi) was used to visualize the conserved motif (motif 2) of 117 *NAC* genes from *E. senticosus* and 135 *NAC* genes from *A. thaliana*. The 2000 bp sequence preceding the starting codon of *EsNAC* genes was captured and analyzed for the genetic elements using PLANTCARE (http//bioinformatics.psb.ugent.be/webtools/plancare/html/). The genes were then classified and visualized using TBtools [14].

### Expression and correlation analyses of *EsNAC* genes

The transcriptome sequencing data of genes with different DNA methylation levels in *E. senticosus* were extracted from NCBI (SRR19962743-SRR19962757) [14]. The consistency between *EsNAC* genes identified from the genome sequencing data and those identified from the transcriptome sequencing data was determined by comparing the two sets of sequencing data using BLAST. Differentially expressed *EsNAC* genes were detected by analyzing the FPKM values obtained from the transcriptome sequencing data. TBtools’ HeatMap function was utilized to visualize the *EsNAC* genes that were expressed differentially. Pearson correlation (PC) analysis was used to evaluate the association between DNA methylation levels and gene expression. Statistical analysis was conducted using the SPSS Statistics software (Version 25.0).

### Validation and analysis of gene expression

For qRT-PCR, the cDNA of *E. senticosus* genes with different methylation levels was used as a template, and GAPDH [14] was used as the internal reference. Supplementary Table 1 displays the PCR primer sequences utilized. The reaction setup and parameters were established according to a prior investigation conducted [8]. The 2^−ΔΔCt^ method [14] was utilized to determine the comparative expression of *EsNAC* genes in every sample. Origin 2022 was used to generate histograms.

The materials used in this study were identified as *E. senticosus*, a plant from the Araliaceae family, by Professor ZhaoBin Xing from the School of Life Sciences at North China University of Science and Technology. The voucher specifications were stored in the laboratory of the School of Life Sciences. The plant material used in the study was *E. senticosus* obtained from biennial cutting propagation from Muling City, Heilongjiang Province, China, and did not require permission. The leaves of *E. senticosus* were used as experimental materials to clone the gene and promoter, and the expression level and saponin content were analyzed.

The *E. senticosus* used in the study was obtained by cuttings and did not require permission. 

### Subcellular localization of *Es*NAC proteins

WoLF PSORT (https //wolfpsort.hgc.jp) was utilized to predict the subcellular localization of *Es*NAC proteins. Based on the transcriptome sequencing data, the open reading frames (ORFs) of differentially expressed *EsNAC* genes associated with DNA methylation were cloned. The PHG-*Es*NAC-GFP recombinant plasmids were constructed and transformed in *Agrobacterium tumefaciens* strain GV3101 and injected into onion epidermal cells separately. Following a 48-hour incubation period, the laser confocal scanning microscope [14] was utilized to identify GFP signals. Supplementary Table 1 displays the primer sequences utilized for the cloning process.

### Effect of DNA methylation on the promoter activity of key enzyme-coding genes

To create pCAMBIA1304-GUS-*EsFPS*, -*EsSS*, and -*EsSE* recombinant plasmids, the promoters of *EsFPS*, *EsSS*, and *EsSE* [8] were inserted into the pCAMBIA-1304 expression vector. Supplementary Table 1 displays the primer sequences utilized for the cloning process. Subsequently, recombinant plasmids with different DNA methylation levels were formed by treating the plasmids with CpG methyltransferase (M. SssI) for 0, 20, 40, and 60 min (New England Biolabs (Beijing) LTD.). Using bisulfite sequencing technology, the methylated promoters *EsFPS*, *EsSS*, and *EsSE* were subjected to bisulfite treatment. Subsequently, the treated DNA was amplified through PCR using methylation-specific PCR kits from TIANGEN Biotech Co., Ltd., located in Beijing, China, along with specific primers. The resulting amplification products were sequenced to validate the successful methylation of the promoters [8]. The recombinant plasmids and empty pCAMBIA1304 plasmid were transformed in *Agrobacterium tumefaciens* strain GV3101, transfected into *N. benthamiana*, and cultured for 3 days. The GUS Staining Kit (Coolaber, Beijing) was utilized for GUS staining, and the optical density was assessed using the Image J software.

### Analysis of transcriptional self-activating activity of *EsNAC* genes

The ORFs of screened *EsNAC* genes were integrated into the pGBKT7 vector using the Seamless Cloning Kit (Beyotime Biotech Inc, Shanghai). Supplementary Table 1 displays the primer sequences utilized for the cloning process. The recombinant plasmids and the control empty pGBKT7 vector were transformed into yeast strain AH109 individually and then cultured on agar plates with SD/-Trp medium at 28 °C for a period of 2 to 3 days. The yeast monoclonal clones that underwent successful transformation with the desired plasmid were chosen and cultivated at a temperature of 28℃ for a period of 48 h. Following this, the monoclonal clones were diluted by a factor of 10 and 100, and 5 µL of both the original solution and the diluted bacterial solution were cultured on agar plates containing SD/-Trp medium and SD/-Trp/-His/-Ade medium for the purpose of screening. The samples were incubated for 2 days at 28℃ until colonies appeared and photographed using a camera [63].

### Expression of *Es*NAC proteins in vitro and analysis of their interaction with DNA

The ORFs of *EsNAC* genes were cloned into the PGEX-4T expression vector using the Seamless Cloning Kit (Beyotime Biotech Inc, Shanghai). Supplementary Table 1 displays the primer sequences utilized for the cloning process. The recombinant plasmids were transformed in E. coli strain BL21 (DE3) to induce the expression of each *EsNAC* gene [64]. The cell precipitates and suspensions underwent separation, followed by lysis in a buffer and centrifugation at 16,000 g for 1 h. The GST-tag Protein Purification Kit (Beyotime Biotech Inc, Shanghai) was used to purify *Es*NAC proteins in the supernatant for gel block analysis [65].

The binding sites of *EsNAC* in *EsFPS*, *EsSS*, and *EsSE* promoters were predicted using JASPAR (https://jaspar.genereg.net/). The corresponding regions were amplified via PCR, recovered, and purified using the Universal DNA Purification Kit (Beijing Biomed Gene Technology Co., Ltd.). Supplementary Table 1 displays the primer sequences employed for PCR. The recovered products were used as probes for EMSA after DNA methylation was induced by CpG methyltransferase (M. SssI) (New England Biolabs Beijing LTD.). Biotin-labeled probes were obtained using the EMSA Probe Biotin Labeling Kit (Beyotime Biotech Inc, Shanghai). In the binding reaction, 10 µL was used, consisting of 1 µL of 10× binding buffer, 2 µg of *Es*NAC protein for each, and 1 µL of the biotin-labeled 5-mC probe. The incubation of the binding reaction system took place at a temperature of 28℃ for a duration of 20 min, followed by separation on a non-denaturing polyacrylamide gel with a concentration of 6%. Following electrophoresis, the gels were moved onto Amersham Hybond-N nylon membranes. The capacity of each responsive *Es*NAC protein to bind to methylated *EsFPS*, *EsSS*, and *EsSE* promoters was assessed by observing the presence or absence of bands after development and fixation [66].

### Prediction of DNA rigidity and molecular docking between *EsNAC* genes and target DNA

BendNet (http://www.dnabendnet.com/) was used to predict the DNA rigidity of *EsFPS*, *EsSS*, and *EsSE* promoters [33]. TFPred (http://lin-group.cn/server/TFPred/) was used to predict whether *Es*NAC could bind to methylated *EsFPS*, *EsSS*, and *EsSE* promoters. Before and after methylation, the *Es*NAC protein sequences were subjected to molecular docking with the *EsFPS*, *EsSSS*, and *EsSE* promoter probe sequences using the HDOCK server (http//hdock.phys.hust.edu.cn/). The results of the docking were visualized using Pymol and Chimerax [64].

### Dual-luciferase reporter assay

Using the Seamless Cloning Kit (Beyotime Biotech Inc, Shanghai), the ORFs of *Es*NAC genes were inserted into the pGreenII 62-SK plasmid as effector plasmids, while the promoters of *EsFPS*, *EsSS*, and *EsSE* [8] genes were inserted into the pGreenII 0800-LUC vector to create recombinant plasmids. Supplementary Table 1 displays the primers employed for cloning. The pGreenII 0800-LUC recombinant plasmids underwent DNA methylation with CpG methyltransferase (M. SssI) from New England Biolabs (Beijing) LTD. and were utilized as reporter plasmids. These plasmids were transformed in *Agrobacterium tumefaciens* strain GV3101. Both effector and reporter plasmids were injected into *N. benthamiana* leaves in a ratio of 1:1 and cultured for 3 days. Tobacco proteins were extracted from a buffer containing a complete protease inhibitor cocktail. The levels of luciferase activity for LUC and REN were assessed utilizing the Dual-Luciferase® Reporter Assay System (Promega Beijing Biotech Co., Ltd.), and the ratio between the two activities was computed [66, 67].

### Effect of overexpression of *NAC* genes on saponin content in *E. senticosus*

 The ORFs of *EsNAC* genes were cloned into the pCAMBIA-1300 overexpression vector using the Seamless Cloning Kit (Beyotime Biotech Inc, Shanghai). Table 1 displays the primer sequences utilized for the cloning process. *Agrobacterium tumefaciens* strain GV3101 was used to transform both the overexpressed recombinant plasmids and the empty plasmids. The same growth state of *E. senticosus* leaves were infiltrated using *Agrobacterium rhizogenes* strain GV3101 and then incubated at a temperature of 28 °C for a period of 2 to 3 days. The expression of *EsNAC*, *EsFPS*, *EsSS*, and *EsSE* genes was detected using qRT-PCR [8, 10]. Total saponins were extracted using a method reported in a previous study [8, 10]. The control substance used was Oleanolic acid from Beijing Solarbio Technology Co., Ltd. The total saponin content was measured using ultra-high-performance liquid chromatography (SHIMADZU Essentia LC-16) under the following conditions: a chromatographic column called Kromasil 100-5-C18 4.6 × 250 mm was used, the column temperature was set at 35℃, methanol was used as the mobile phase, the flow rate was 0.5 mL/min, and the detection wavelength was 210 nm. A standard curve *y* = 2 × 10^8^*x*+2500.9, *R*^2^ = 0.9923 of peak area and oleanolic acid concentration was established using the external standard method. In the curve, x represented saponin content and y represented the peak area. The concentration of oleanolic acid was calculated based on the peak area and used to replace the total saponin content of the sample [1, 68, 69].

### Supplementary Information

Supplementary Material 1.

Supplementary Material 2.

Supplementary Material 3.

Supplementary Material 4.

## Data Availability

All data generated or analyzed during this study are included in this published article, its supplementary information files and publicly available repositories. The sequencing data generated in this study have been deposited in the National Center for Biotechnology Information (NCBI) under the accession number SRX13417593-SRX13417601 [14].
