# Supplementary material for: Identification of Eleutherococcus senticosus NAC transcription factors and their mechanisms in mediating DNA methylation of EsFPS, EsSS, and EsSE promoters to regulate saponin synthesis
Source: BMC Genomics. 2024 May 31;25:536. doi: 10.1186/s12864-024-10442-8 (PMC11140872; doi:10.1186/s12864-024-10442-8)
Supplement: Supplementary file 4 — Supplementary Material 4. [file 12864_2024_10442_MOESM4_ESM.docx]

**Supplementary figure 1 ：Molecular docking of *Es*NAC to promoters.**


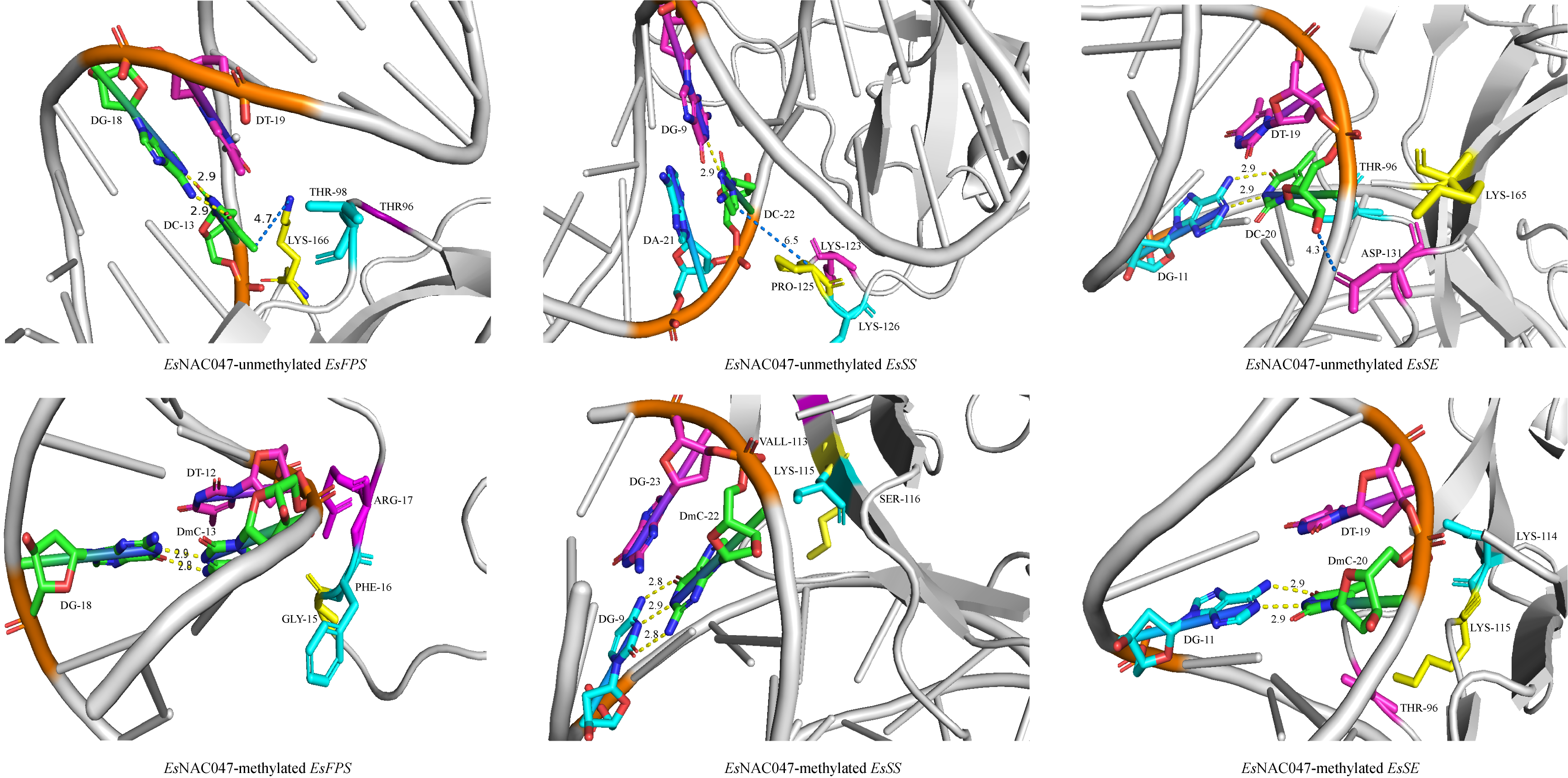


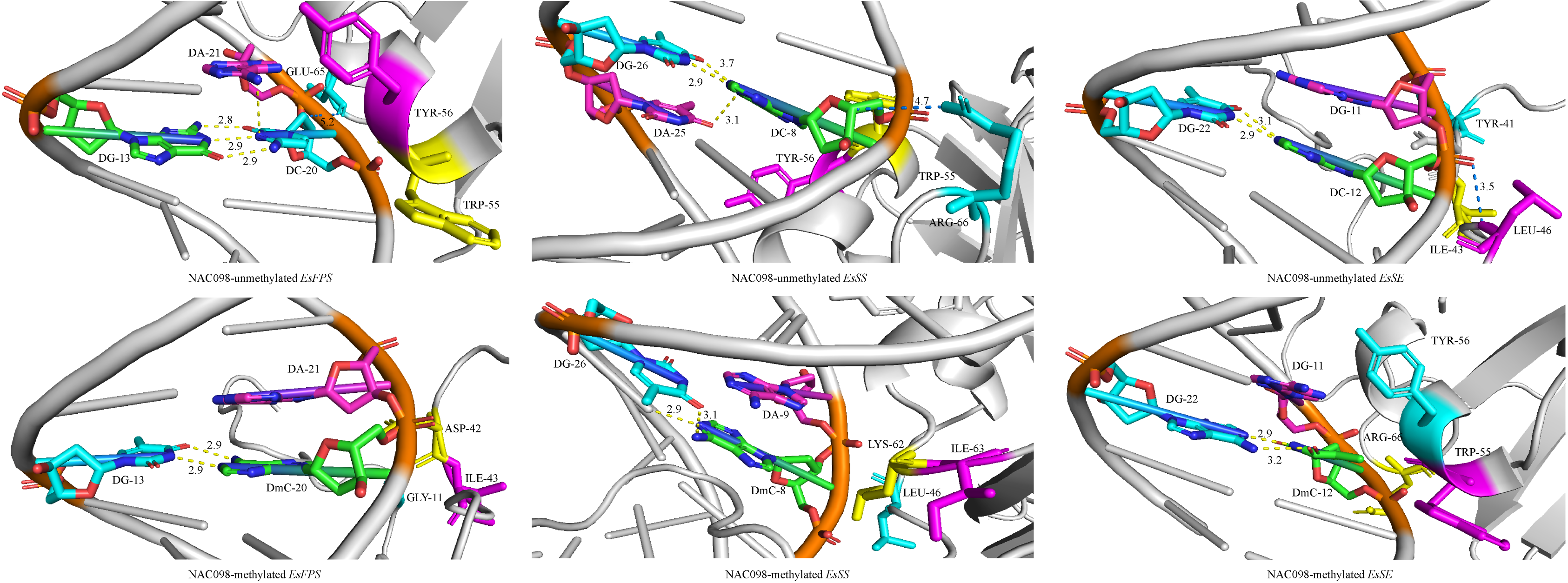


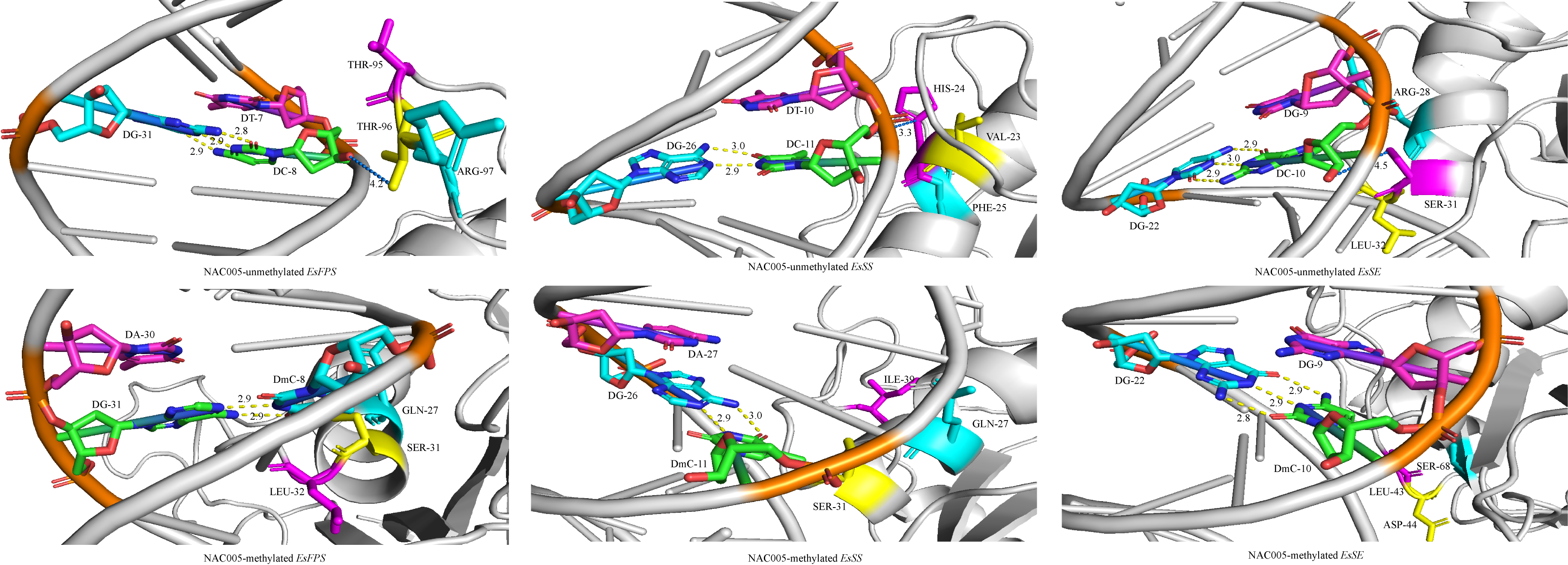


Molecular docking of *Es*NAC047, *Es*NAC098, and *Es*NAC005 with *Es*FPS, *Es*SS and *EsSE* promoters of different methylation degree. Note: Blue dotted lines are the docked hydrogen bonds of the binding sites, yellow dotted lines are the undocked hydrogen bonds, and the number next to the hydrogen bond is the bond length.
